# Biomarkers for the Noninvasive Diagnosis of Endometriosis: State of the Art and Future Perspectives

**DOI:** 10.3390/ijms21051750

**Published:** 2020-03-04

**Authors:** Costin Vlad Anastasiu, Marius Alexandru Moga, Andrea Elena Neculau, Andreea Bălan, Ioan Scârneciu, Roxana Maria Dragomir, Ana-Maria Dull, Liana-Maria Chicea

**Affiliations:** 1 Department of Medical and Surgical Specialties, Faculty of Medicine, Transilvania University of Brasov, 500019 Brasov, Romania; canastasiu@gmail.com (C.V.A.); moga.og@gmail.com (M.A.M.); urologie_scarneciu@yahoo.com (I.S.); roxana.gidinceanu@unitbv.ro (R.M.D.); 2 Department of Fundamental, Prophylactic and Clinical Sciences, Faculty of Medicine, University Transilvania Brasov, 500019 Brasov, Romania; 3 Regina Maria Hospital, 500091 Brasov, Romania; dullana2005@yahoo.com; 4 “Victor Papilian” Medical School, University “Lucian Blaga” of Sibiu, 550024 Sibiu, Romania; liana.chicea@gmail.com

**Keywords:** biomarker, angiogenesis, cytokines, urinary biomarkers, endometriosis

## Abstract

Background: Early and accurate diagnosis of endometriosis is crucial for the management of this benign, yet debilitating pathology. Despite the advances of modern medicine, there is no common ground regarding the pathophysiology of this disease as it continues to affect the quality of life of millions of women of reproductive age. The lack of specific symptoms often determines a belated diagnosis. The gold standard remains invasive, surgery followed by a histopathological exam. A biomarker or a panel of biomarkers is easy to measure, usually noninvasive, and could benefit the clinician in both diagnosing and monitoring the treatment response. Several studies have advanced the idea of biomarkers for endometriosis, thereby circumventing unnecessary invasive techniques. Our paper aims at harmonizing the results of these studies in the search of promising perspectives on early diagnosis. Methods: We selected the papers from Google Academic, PubMed, and CrossRef and reviewed recent articles from the literature, aiming to evaluate the effectiveness of various putative serum and urinary biomarkers for endometriosis. Results: The majority of studies focused on a panel of biomarkers, rather than a single biomarker and were unable to identify a single biomolecule or a panel of biomarkers with sufficient specificity and sensitivity in endometriosis. Conclusion: Noninvasive biomarkers, proteomics, genomics, and miRNA microarray may aid the diagnosis, but further research on larger datasets along with a better understanding of the pathophysiologic mechanisms are needed.

## 1. Introduction

Endometriosis is considered a debilitating gynecological pathology with a high prevalence among young women [1]. The incidence of the disease varies between 6–10% [2]. Various sources indicate a constant increase in the number of cases, reaching almost 15% worldwide [3]. Endometriosis is characterized by the migration of endometrial-like cells in ectopic places outside the uterus. The clinical manifestations thereof consist of chronic pelvic pain, dysmenorrhea, and infertility, the latter being reported in 30–50% of cases, while 20–25% of patients remain asymptomatic [4].

Given the nonspecific symptoms of this disease that can usually mimic those associated with pelvic inflammatory disease or other conditions associated with chronic pelvic pain, the gold standard for a definitive diagnosis consists of surgical procedures, followed by histopathological exams [5]. Under these circumstances, a considerable diagnostic delay is explicable [6,7], leading to 8–12 years of belated appropriate treatment [8]. As of yet, reliable laboratory biomarkers for this gynecological pathology remain elusive. The increased incidence of this pathology in women with early menarche compels the development of novel noninvasive diagnostic biomarkers for faster diagnosis, appropriate treatment, and for triaging potential patients for surgery [9,10]. A biomarker is a biological molecule that can be “objectively measured and evaluated as an indicator of normal biological processes, pathogenic processes, or pharmacological responses to a therapeutic intervention” [11]. Therefore, a biomarker or a panel of biomarkers found in the biological fluids of the affected women could be an expedient diagnostic tool for endometriosis as well as an objective assessment of the effectiveness of the treatment [12].

The pathophysiology of this disease is not thoroughly understood. Sampson’s theory of retrograde menstruation is still viewed as the principal etiopathogenic factor of endometriosis. However, approximately 90% of non-affected women undergo retrograde menstruation [13]. Novel pieces of evidence support the hypothesis that endometriosis development is caused by the occurrence of primitive endometrial cells outside the uterus, during organogenesis. Following puberty, these cells differentiate into functional endometriotic implants [14]. Other authors suggested that the principal source of extrapelvic endometriosis is represented by bone marrow-derived stem cells, which are able to migrate through the peripheral circulation and induce endometriosis in remote sites [15].

According to the “embryonic theory” or epigenetic theory, during organogenesis, the genes of the Homeobox and Wingless family are essential for the differentiation of the anatomical structures of the urogenital tract. Any dysregulation of these genes through the Wnt/b-catenin signaling pathway will lead to various anomalies and may cause aberrant placement of the stem cells. The abnormal placement of these cells, associated with immune alterations and the pro-inflammatory peritoneal environment, will determine the progression towards endometriosis [14]. An ectopic endometrium displays a distinctive epigenetic expression profile, which involves homeobox A (HOXA) clusters and Wnt signaling pathway genes [16]. Furthermore, miRNAs dysregulations were found to modulate the proliferation and invasiveness of ectopic endometrial cells. Eggers et al. [17] pointed out that dysregulation of miR-200b family affects the differentiation of ectopic cells by regulating epithelial-to-mesenchymal transition. Moreover, epigenetics plays an important indirect role in the recruitment and differentiation of bone marrow-derived stem cells by modulating the relationship between the inflammatory microenvironment and steroid action. This interaction represents the trigger for the recruitment of bone marrow-derived stem cells, and it is highly influenced by the epigenetic expression profile [16].

Endometriosis is considered an inflammatory disease, due to increased levels of activated macrophages and cytokines such as interleukins (IL-6, IL-8, IIL-1β), tumor necrosis factor-alpha (TNF-α), and macrophage migration inhibitory factor (MIF), in the peritoneal fluid of affected women [2]. Furthermore, several inflammatory biomarkers registered increased levels in the serum of endometriotic women: C-reactive protein (CRP), IL-4, TNF-α, monocyte chemoattractant protein-1 (MCP-1), IL-6, IL-8, and regulated on activation, normal T cell expressed and secreted (RANTES) [13].

Recently, a wide range of papers revealed the importance and effectiveness of various putative biomarkers from the biological fluids of affected patients. Despite considerable research on this topic, noninvasive biomarkers of endometriosis have eluded the transition from bench to bedside. Some of the limitations of biomarker studies consist of reduced datasets, methodological flaws (the variability of biomarkers under physiological conditions such as menstrual phases), the lack of reproducibility across multiple studies, and, last but not least, high costs of the complex assays.

**Figure 1:**
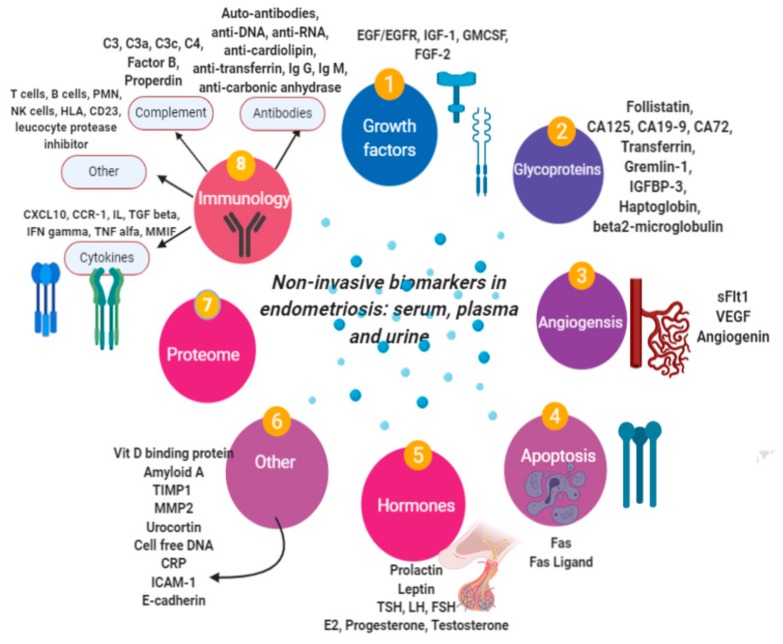
Putative noninvasive biomarkers for endometriosis [12].

This paper aims to synthesize the expanding body of knowledge regarding the putative noninvasive biomarkers (Figure 1) of endometriosis. Furthermore, it presents the opportunities offered by the newest technologies such as proteomics, genomics, and miRNA microarray, which may represent the future perspective of diagnosis.

## 2. Material and Methods

This study is a synthesis of noninvasive biomarkers of endometriosis, based on literature review, highlighting their possible use for early and precise diagnosis while emphasizing the newest perspectives and opportunities in the diagnostic field.

Our research included all the publications in Google Scholar, PubMed and Cross Ref related to noninvasive biomarkers for endometriosis, during the period January 2000 to December 2019, using the following Medical Subject Headings (MeSH) keywords: biomarker, angiogenesis, cytokine, urinary biomarkers, and endometriosis. Two authors independently identified and selected relevant articles based on the following inclusion criteria: full-text articles, written in English, human-based studies, biomarkers retrieved from serum, plasma, or urine. All the studies included patients and controls that have either tested positive or negative for endometriosis. The exclusion criteria were, as follows: studies on animals or involving cell cultures, biomarkers retrieved from any invasive procedure (endometriotic tissue, endometriomas content or peritoneal fluid), papers written in other languages than English, duplicate papers, abstracts and papers that only compared biomarker levels between affected women with different stages of endometriosis, without the inclusion of healthy controls. A total number of 55 studies met our criteria.

## 3. Endometriosis—An Inflammatory Disease

A thorough understanding of the pathogenic and molecular mechanisms of endometriosis represents the springboard in finding various molecules that could serve as noninvasive biomarkers for early diagnosis.

Endometriosis is characterized by chronic abdominal pain, infertility, dysmenorrhea, and sometimes anxiety, related to the degree of the pelvic pain [18,19]. This pathology is the result of interactions between various hormonal, immunological, and genetic factors, and it is characterized by an increased expression of inflammatory factors and angiogenesis [20].

The central role in endometriosis belongs to local inflammation, which triggers the development of the disease and induces pain and infertility [21]. In support of this statement, a wide range of studies reported that the peritoneal fluid of endometriotic patients contained high levels of macrophages and immune cells, which secrete cytokines, angiogenic factors, and growth factors [22]. A further factor influencing the expression of these cytokines and of other cell adhesion molecules is represented by oxidative stress, thought to be an associated compound of endometriosis-related inflammation [23].

Normally, the peritoneal fluid contains 85% macrophages, as well as mast cells, lymphocytes, and mesothelial cells [24]. During menstruation and in endometriosis, this percentage is increased, and macrophages are more able to release prostaglandins (PG), cytokines, complement components, hydrolytic enzymes, and growth factors, which are central contributors to the endometriosis pathogenesis [25], and thus, possible biomarkers for the early diagnosis of this pathology. Since elevated values of PGs have been discovered in the peritoneal fluid of affected women, it is considered that PGs can trigger the progression of the disease by their inhibitory action through macrophage function, and their capacity to increase cellular proliferation and angiogenesis rate [25].

The inflammatory environment of this disease points out an increased production of estrogens, which in turn will raise the production of PGs through the activation of both NF-kB and cyclooxygenase-2 (COX-2) [26]. A paper by Banu et al. highlighted the influence of COX-2 and prostaglangin E2 (PGE2) on the migration and invasion of endometrial cells. Furthermore, the inhibition of COX-2 was able to decrease the invasion of epithelial and stromal cells in endometriosis [27].

COX-2 usually presents high values in ectopic endometrioid implants. Some studies found increased COX-2 expression and increased COX-2-derived PGE2 production in ectopic implants [27]. PGE2 and other proinflammatory cytokines induce increased values of COX-2 in normal and ectopic endometrial cells [28]. In ectopic endometriotic stromal cells, proinflammatory cytokines, such as interleukine-1β (IL-1β), are able to increase mRNA stability and to upregulate COX-2 promoter activity. This positive regulation of COX-2 is achieved via mitogen-activated protein kinase (MAPK)-dependent signaling pathways, which facilitate the binding of COX-2 promoter to the cAMP-responding element site. The subsequent dysregulation of MAPK signaling pathways increases inflammation, thereby recruiting immune cells and amplifying the inflammatory response [29].

Human endometrial cells have been shown to express NF-kappa B subunits [30,31], which are activated during normal menstruation [32]. In vitro, the activation of NF-kappa B in endometriotic stromal cells has proven a positive modulation of the proinflammatory cytokines, interleukins (IL-8, IL-6), RANTES, TNF-alpha, macrophage migration inhibitory factor (MIF), MCP-1, intercellular adhesion molecule (ICAM)-1, and granulocyte-macrophage colony-stimulating factor (GM-CSF) [33]. Alternatively, in vivo, the integrative activation of NF-kappa B subunits has been highlighted in ectopic areas of endometrial tissue [34]. The researcher’s common point of view is that in endometriosis, NF-kappa B activated macrophages release IL-1, 6, 8, COX-2, TNF-alpha, and vascular endothelial growth factor (VEGF); thus, perpetuating the inflammatory pathway [21].

Several studies also suggested the contribution of oxidative stress in endometriosis-associated inflammation. Increased production of reactive oxygen species (ROS) inside the peritoneal environment may promote the inflammatory process through the positive regulation of several proinflammatory genes [35]. Zeller et al. [36] reported increased production of ROS into the peritoneal fluid of affected women. Portz and collab. [37] suggested that injecting antioxidant enzymes into the peritoneal cavity of endometriotic subjects could prevent the formation of intraperitoneal adhesions. However, other researchers, which directly measured the production of ROS in the peritoneal cavity of the affected patients did not find any obvious oxidant or antioxidant imbalance [38,39]

## 4. Immunological Aspects of Endometriosis

Immune dysfunction characterized by hyperactive peritoneal macrophages with altered phagocytic ability represents another key-point in endometriosis development and progression. The phagocytic ability of macrophages is mediated by matrix metalloproteinases (MMP), enzymes capable of destroying the structure of the extracellular matrix, and by the expression of several macrophage surface receptors, that can promote the dissolution of cellular debris [40]. Lagana et al. [41] suggested that macrophages are classified into M1 macrophages, and M2 macrophages, which display anti-inflammatory and pro-fibrotic activities. Moreover, these molecules are able to induce immunotolerance. In their study, Lagana and coworkers collected tissues from the ovarian endometriomas of women at different stages of endometriosis and measured M1 and M2 macrophage levels. They observed that the number of both M1 and M2 macrophages was significantly higher in the endometriosis group compared to controls. Furthermore, a progressive decrease of M1 macrophages and an increase of M2 macrophages from stage I to stage IV endometriosis was also noted [41].

Tie-2-expressing macrophages is a subset of macrophages that have been proved to promote tumor growth and angiogenesis. Using a murine model, these molecules were observed surrounding recently developed endometriotic blood vessels [42]. Should this hypothesis be proven accurate, it would infer the progression from endometriosis to ovarian cancer, thus explaining the increased risk of ovarian cancer in women affected by endometriosis [43].

Natural Killer cells (NK) activity is usually significantly decreased in endometriosis patients. This immunosuppressive effect, which consists of the reduction of T cell-mediated cytotoxicity, is not related to steroid hormone levels or to the day of the menstrual period. Wu et al. [44] explained this low activity of NK cells through highly increased levels of killer cell inhibitory receptors (KIRs) on NK cells of women with stage III-IV endometriosis. Recently, a new subset of T cells was identified, Invariant Natural Killer T cells (iNKT). They combine both classically innate and adaptive immunologic characteristics. These molecules are able to secrete both Th1 and Th2 cytokine patterns, and their actions in the eutopic and ectopic endometrium are subject to further research [45].

The contribution of IL-10 to endometriosis has also been suggested. Elevated levels of this cytokine-induced a decreased activity of cytotoxic T lymphocytes and CD+ helper cells into the peritoneal fluid of affected patients [46]. Furthermore, M1 to M2 macrophage polarization is promoted by the increased expression of IL-10 [47].

Several studies were conducted on transgenic mice in order to asses the levels of mucin 1 (MUC1) and Foxp3+ CD4 lymphocytes (Tregs). Under normal conditions, MUC1 is present on eutopic endometrial glands but is overexpressed in ectopic lesions. Preclinical investigations have revealed that upon disease development, endometriotic mice displayed high titers of anti-MUC1 antibodies and increased levels of Tregs [48]. These findings suggest that Treg lymphocytes could represent a promissing venue for additional research.

Further immunological factors involved in the development of endometriosis are represented by T helper cells (Th), IL-17A and IL-4, as showed by Osuga et al. [49]. They observed increased levels of Th2 and Th17 cells in endometriotic tissues. IL-4 stimulates the proliferation of endometriotic stromal cells and IL-17A induces neutrophil migration in endometriotic tissues. Moreover, IL-17A, combined with TNFα, enhances the secretion of IL-8 and CCL-20, suggesting the cooperation of inflammation and Th17 immune response. Given the body of scientific proof, these molecules could be the focus of further reasearch for endometriosis treatment as well as potentially viable biomarkers for the disease development.

Since endometriosis is now considered an immune dysregulation with profound changes in the activities of various cells involved in immune reactions, further studies might offer novel therapeutic targets as well as biomarkers for early detection of endometriosis.

## 5. Angiogenesis in Endometriosis

Endometriosis is a polygenic and multifactorial disease, and increased angiogenesis and proteolysis may trigger its development and progression [50]. Studies have shown an essential involvement of several angiogenesis-related factors in endometriosis, such as the Delta-like 4 (Dll4)-Notch signal pathways, angiopoietin, vascular endothelial growth factor (VEGF), and vascular endothelial growth factor receptor (VEGFR) [51].

Vascular endothelial growth factor (VEGF), and vascular endothelial growth factor receptor (VEGFR) are the most well-known molecules involved in the angiogenesis process. They are able to regulate the proliferation, migration, and permeability of the cells. A study by Ahn et al. [52] pointed out that IL-17A is emerging as a potent angiogenic factor, and it can upregulate VEGF and IL-8. This mechanism promotes intraperitoneal angiogenesis in order to maintain the ectopic foci and to facilitate the development of new ones. Furthermore, plasmatic concentrations of IL-17A displayed a significant decrease following surgical removal of ectopic endometrial tissue.

The expression of VEGF microRNA (mRNA) is more increased in hypoxic areas. The growth of endometriotic cells creates hypoxia, enhancing the production of several molecules with pro-angiogenic potentials, such as TNF-α, VEGF, IL-8, bFGF, and TGF-β. All these factors trigger vessel hyperpermeability, release plasmatic proteins, induce the formation of fibrin, endothelial cells proliferation, and promote angiogenesis and fibrinolysis [53].

Several studies reported that interleukins also display a pro-angiogenic effect. Interleukins with a Glutamic Acid-Leucine-Arginine-amino-terminal site, such as IL-8, increase the angiogenesis rate, while those lacking this sequence, such as IL-4, impede this process [54]. Volpert et al. [55] highlighted that in vivo, IL-4 inhibits the vessel’s neoformation induced by bFGF, while in vitro, it impedes the transmigration of endothelial cells towards bFGF. Another interleukin involved in endometriosis-related angiogenesis is IL-1α, which promotes this process through the augmented expression of various angiogenic factors such as VEGF, IL-8, and bFGF, according to the results published by Torisu and collab [56].

Donnez et al. [57] highlighted that red and white endometriosis foci exhibit different degrees of expression of pro-angiogenic factors. Consequently, they have shown that red endometriosis lesions display a more pronounced vascularization and a higher cell proliferative activity.

**Figure 2:**
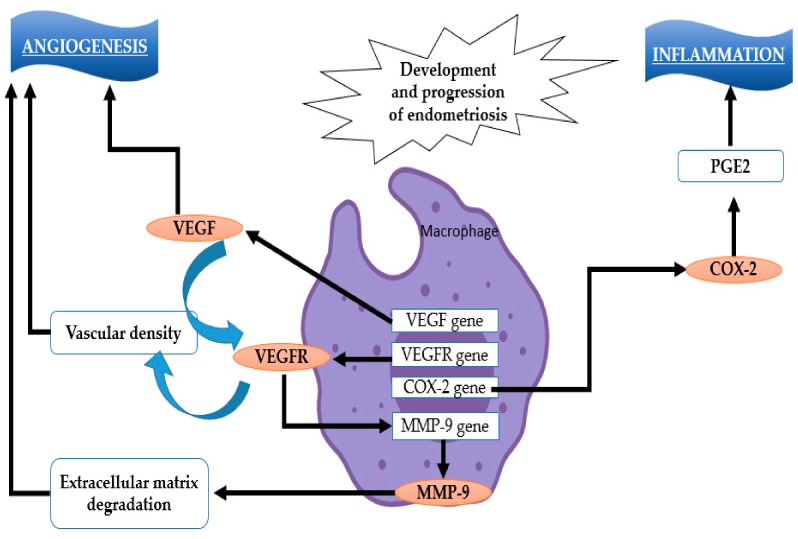
Angiogenesis process—In endometriosis, the central role in pathophysiological mechanisms is attributed to macrophages. These cells increase the intranuclear expression of vascular endothelial growth factor (VEGF) and cyclooxigenase 2 (COX-2) genes, leading to increased angiogenesis and an inflammatory environment [58].

Figure 2 is a schematic representation of the mechanism of development and progression of endometriosis, illustrating the inflammatory and angiogenetic pathways.

## 6. Studies of New Noninvasive Biomarkers in Endometriosis

A noninvasive biomolecule for endometriosis diagnosis could be extracted and quantified from serum, plasma, or urine and would be beneficial for patients with chronic pelvic pain, infertility, and dysmenorrhea, in the context of a regular ultrasound.

Blood and urine make for excellent sources of biomarkers due to their reproducibility, ease of access, and measurement [59]. Promising potential endometriosis biomarkers that underwent testing were: glycoproteins, growth factors, miRNAs, lncRNAs, as well as proteins related to the angiogenesis process or immunology [12]. Despite extensive research, neither a single biomarker nor a panel of biomolecules has been considered sufficiently specific and sensitive to be used as a diagnostic test for endometriosis [12].

### 6.1. Serum/Plasma Biomarkers of Endometriosis

#### 6.1.1. Glycoproteins

Although lacking both specificity and sensitivity for this pathology, the most representative glycoprotein used as a biomarker for endometriosis is CA-125 [60]. However, the simultaneous measurement of CA-125 with other molecules and their combination showed different sensitivities and specificities for endometriosis. For example, Mihalyi et al. combined CA-125 with IL-8 and TNF-α during the secretory phase of the menstrual cycle and observed that this combination gives a sensitivity of 89.7% and specificity of 71.1% for endometriosis [61]. Furthermore, the combination of CA-125, chemokine receptor (CCR) type1, mRNA, and MCP1 proved to be efficient as a biomarker panel, with a sensitivity of 92.2% and specificity of 81.6%, according to Agic et al. [62]. Vodolazkaia combined four biomarkers, namely: VEGF, CA-125, Annexin V, and glycodelin resulting in a sensitivity and a specificity of 74–94% and 55–75%, respectively [63].

Another tumor marker, CA-19-9, was investigated as a possible biomarker for endometriosis diagnosis. Elevated values of this glycoprotein were detected in endometriotic women in comparison to healthy patients, but the sensitivity of this molecule was significantly lower when compared to CA-125 [12]. It is worthwhile mentioning that CA-19-9 has shown a positive correlation with the severity of the disease [64].

Glycodelin is a glycoprotein that promotes cell proliferation and neovascularization. Serum levels of this molecule have been found higher in endometriotic women when compared to non-affected patients. Mosbah et al. conducted a study that included women 21–48 years old with endometriosis and measured the levels of intercellular adhesion molecule 2 (ICAM-2), IL-6, and glycodelin A in their serum samples. Their results showed that IL-6 and glycodelin A displayed higher serum as well as peritoneal fluid levels in affected subjects compared to controls, with a sensitivity and specificity of 91.7% and 75.0% for serum glycodelin A, 93.8% and 80.0% for serum IL-6, 58.3%, and 60.0%, respectively for ICAM-1 [65]. Kocbek et al. [66] also identified significantly increased concentrations of glycodelin-A in the serum of cases compared to controls. Furthermore, combinations that included leptin/glycodelin-A ratio and ficolin 2/glycodelin-A ratio displayed a sensitivity and specificity of 72.5% to 84.2% and 78.4% to 91.2%, respectively.

Prentice Crapper [67] analyzed eight putative serum biomarkers for endometriosis, and they observed a significant elevation of only two of these molecules. Its results indicated that glycodelin has a sensitivity and specificity of 81.6% and 69.6%, respectively, for disease diagnosis. Zinc alpha 2-glycoprotein also registered increased values in affected women, with a sensitivity of 46% and a specificity of 100%. The combination of these two markers showed a greater specificity and sensitivity for the detection of this gynecological disorder (65% and 90%, respectively).

Follistatin is an inhibitor of activin, produced by the endometrium. It is increased in endometriosis, and thus, it could be used as a noninvasive biomarker for endometriosis diagnosis. Reis et al. simultaneously evaluated the levels of serum activin A and follistatin in healthy and affected subjects (with ovarian endometrioma, peritoneal endometriosis, and deep infiltrating endometriosis). Their study concluded that follistatin and activin A are not significantly modified in peritoneal or deep infiltrating endometriosis, but their serum concentrations in ovarian endometrioma were slightly higher [68].

Intercellular adhesion molecule-1 (ICAM-1) is a glycoprotein that seems to be involved in the development and promotion of endometriosis. It plays an essential role in the promotion of the inflammatory and immunological reactions, and recent studies associated the polymorphism of the ICAM-1 gene with the severity of this gynecological disorder. Vigano et al. [69] analyzed two polymorphic sites of the ICAM-1 gene, known as G/R241 and E/K469, using a cohort of 188 women with confirmed endometriosis and 175 controls. They observed that the frequency of the R241 allele was slightly higher in the endometriosis cohort than in the control group, while the frequency of the second allele was approximately the same in both groups. In conclusion, genetic polymorphism in the ICAM-1 gene domain G/R241 offers a promising perspective in the field of noninvasive biomarkers. Furthermore, concomitantly with the development of genomics, this glycoprotein could prove conducive in diagnosing endometriosis, as well as in predicting the degree of severity of this pathology.

#### 6.1.2. Inflammatory Cytokines and Immunological Molecules

In the last decades, immunological molecules and inflammatory cytokines have been extensively studied for their potential as noninvasive biomarkers for endometriosis, with the most representative being IL-1, IL-6, IL-8, interferon-γ (IFN-γ), MCP-1, and TNF-α. Othman et al. tested three serum cytokines, both separately and in various combinations. They collected blood samples and measured cytokines levels with the Bio-Plex Protein Array System. The final results concluded that Il-6, MCP-1, and IFN-γ were significantly increased in the serum of affected women, in comparison with non-affected subjects. No difference has been found between serum concentration levels of tumor necrosis factor-alpha (TNF-α), or granulocyte macrophage colony-stimulating factor (GM-CSF). IL-2, IL-8 and, IL-15 were undetectable both in endometriosis and healthy group [70]. Borrelli et al. determined increased levels of IL-8, MCP-1, and RANTES in the peripheral blood of affected patients vs. controls, in 46.1%, 50%, and 75%, of the assessed cases, suggesting their potential use as noninvasive biomarkers for endometriosis [71].

Interleukin 6 is a proinflammatory cytokine that has shown increased serum levels in affected women. A study of Martinez et al. [72] pointed out that patients with Stage I-II endometriosis had increased levels of IL-6, with a sensitivity of approximately 75% and a specificity of 83.3%. In 2010, Socolov et al. [73] included 24 cases of endometriosis and 24 controls in a study and investigated their serum levels of IL-6, IL-8, IL-1, CA-125, and TNF. Their results indicated that IL-6 was above the cut-off threshold of 2 pg/mL in 71% of the cases and 87% of controls, with a sensitivity and specificity of 71% and 12%, respectively. They concluded that the difference between the two groups was not statistically significant.

Interleukin-8 is a chemokine derived from monocyte/macrophage that has also been considered for the noninvasive diagnosis of endometriosis. While some studies found no differences between cases and controls in terms of IL-8 levels [74]. Pizzo et al. [75] pointed out that this interleukin has increased levels in affected women with Stage I–II endometriosis and even higher levels in endometriomas [76].

Tumor necrosis factor alpha (TNF-α) is a proinflammatory cytokine with pro-angiogenic potential. Its role as a biomarker for endometriosis is controversial; while several studies proved increased serum levels of TNF-α in women with endometriosis [77,78,79], others showed no difference between affected patients and healthy controls [70,80]. Cho et al. [79] concluded that TNF-α increases only in the serum of endometriotic women, while urinary levels remain unchanged. The severity of endometriosis has also been associated with elevated serum TNF-α levels [75]. A recent study conducted by Steff et al. [81] revealed that the level of soluble TNF receptor in affected patients registered a significant increase during the follicular phase of the menstrual cycle.

Choi et al. [82] collected serum samples from 50 patients with endometriosis and 35 healthy individuals and used enzyme-linked immunosorbent assay (ELISA) in order to measure the levels of IL-32, 6, 10, 1β, TNF-α, and CA-125. Only IL-32 displayed significantly higher levels in patients compared to controls. When IL-32 was associated with CA-125, the specificity and sensitivity of this combination reached 60% and 82.9%, respectively, thereby suggesting IL-32 as a potential biomarker for endometriosis diagnosis.

Several studies have also assessed Natural killer (NK) cells as potentially viable biomarkers. Kikuchi et al. [83] reported declining levels in the subset of NK cells (CD57 + CD16) in endometriosis, followed by significantly elevated levels of these cells one month following surgery.

Copeptin is a molecule that has augmented values in inflammatory conditions. Tuten et al. [84] determined the serum levels of copeptin, CA-125, C-reactive protein, CA-15-3, and CA-19-9 in a study on 50 women laparoscopically diagnosed with endometriosis and 36 controls. Their results indicated that the level of copeptin was significantly higher in affected women in comparison to controls. Furthermore, these levels were positively correlated with the stage of the disease. However, there is no consensus indicating that cytokines are suitable for early-stage detection of endometriosis, or discriminating patients with endometriosis from patients with other pelvic pathologies.

#### 6.1.3. Oxidative Stress Markers

Despite being still inconclusive, the pathophysiology of endometriosis seems to be based on several theories, including the imbalance between reactive oxygen species (ROS) and antioxidants [85]. This causes an inflammatory response in the peritoneal cavity and ROS to modulate the proliferation of the endometriotic cells [86].

Malondialdehyde (MDA) is a lipid peroxidase which can be considered an oxidative stress marker. A study conducted by Nasiri et al. [87] concluded that, in the serum of endometriotic patients, there are higher values of MDA in comparison with healthy individuals. Furthermore, women with endometriosis have higher levels of lipid hydroperoxides [88], vitamin E [89], and catalase [90], without any conclusive explanation yet.

Superoxide dismutase (SOD) is an enzyme involved in oxidative stress. It registered diminished activity in the plasma of affected patients, supporting the theory of a decreased antioxidant capacity in endometriosis [91]. Andrisani and coworkers [92] studied the activity of carbonic anhydrase in endometriotic women, in comparison to healthy controls. They observed increased enzymatic activity in response to oxidative stress, along with a cytosolic decrease of glutathione content in affected patients.

The major limitations of all the above-mentioned research papers consist of interlaboratory variations. As such, in order to compare the results across different studies, an all-encompassing methodology is needed.

#### 6.1.4. Growth Factors and Peptides

Our research revealed a scarce number of studies conducted on growth factors and peptides, with a large body of uncertainty and controversy regarding their possible use as biomarkers.

Insulin-like growth factor-1 (IGF-1) has been increased in stages III–IV of endometriosis, but not in stages I–II [93]. However, Steff et al. [94] revealed no statistically significant difference between patients and controls in terms of IGF-1. IGFBP3 is a protein that ensures the transport of IGF-1 and is involved in endometrial cell growth. There are two studies that have proven no difference between serum levels of IGFBP3 in healthy women compared to endometriotic patients [93,95].

Nesfatin-1 was initially described as a hypothalamic neuropeptide, with the ability to lower the intake of food [96]. Sengul et al. [97] investigated the level of serum nesfatin-1 in women with endometriosis, before and after the adjustment for body mass index (BMI). At the end of the study, their results indicated that serum nesfatin-1 levels were significantly decreased in the group of subjects, compared to healthy controls, with unchanged results following BMI adjustment.

Urocortin is a neuropeptide that can be found in both endometrium and ovary, and several research papers highlighted the similarity of serum urocortin levels in endometriotic subjects and healthy controls [98,99]. A further study indicated that serum levels of urocortin were significantly increased in women with ovarian endometriosis in comparison to women with other benign ovarian cysts with a sensitivity of 88% and specificity of 90% [100].

Leptin is a helical cytokine that regulates the process of steroidogenesis as well as the decidual transformation of the endometrium. Chmaj-Wierzchowska et al. [99] revealed that the serum level of this molecule was slightly lower in women with ovarian endometriomas compared to control. However, these differences were not statistically significant.

#### 6.1.5. Angiogenesis Molecules

While some studies suggested that VEGF-A levels following laparoscopic excision of endometriosis foci are reduced [101,102], Szubert et al. [103] concluded that following danazol treatment, plasmatic VEGF concentrations were significantly increased, therefore implying that this molecule could not be associated with the disease.

Pigment epithelium-derived factor (PEDF) is an inhibitor of angiogenesis with neurotrophic and anti-inflammatory properties, and modified values of this molecule have been found in endometriotic patients. Chen et al. [104] analyzed the serum levels of PEDF in 43 women with laparoscopically confirmed endometriosis and 28 controls, using the enzyme-linked immunosorbent assay. The results of their study indicated that PEDF was significantly decreased (16.3 ± 6.6 ng/mL) in cases compared to controls (24.5 ± 7.3 ng/mL) and was correlated with the severity of the symptoms.

While elevated values of serum fibroblast growth factor-2 (FGF-2), and angiogenin have also been recorded in the quest for viable biomarkers, soluble endothelial growth factor (EGF) and platelet-derived growth factor (PDGF) revealed no sizable differences between cases and controls; therefore, being sidelined as potential candidates [12].

#### 6.1.6. Autoantibodies

In the last years, several authors focused on the role of circulating antibodies that could be involved in the pathogenesis of endometriosis. The close interrelation of the endometrium with the immune system justifies and partly explains the mechanism of development, as well as the progression of endometriosis.

Anti-endometrial antibodies and total immunoglobulin have also been considered as noninvasive biomarkers for endometriosis. The antibodies with the highest potential as biomarkers for this pelvic pathology were antibodies against α2-HS glycoprotein, malondialdehyde-modified low-density lipoprotein, laminin-I, lipid peroxide, modified rabbit serum albumin, cardiolipin, and specific antibodies against carbonic anhydrase and transferrin [12]. Ozhan et al. [105] researched serum syntaxin-5, anti-endometrial antibody, and other molecules as noninvasive biomarkers. They concluded that the blood serum levels of syntaxin-5 were significantly increased in the endometriosis group, compared to the control group. Furthermore, serum levels of syntaxin-5 in stage I and II endometriosis were found to have different levels compared to controls.

Gajbhiye et al. [106] included 40 endometriosis patients in their study and noted higher serum levels of autoantibodies compared to tropomodulin 3 (TMOD3), tropomyosin 3 (TPM3), and stomatin-like protein 2 (SLP2) in contrast to control’s serum samples. Elevated values were associated with both minimal to mild and moderate to severe disease. Additionally, in women with endometriomas, autoantibodies against IGF-2 mRNA-binding protein 1 (IMP1) were identified by Yi et al. to be significantly elevated compared to healthy controls [107].

Anti-α-enolase antibodies in endometriotic subjects were significantly elevated compared to non-affected subjects. The sensitivity and specificity of serum Anti-α-enolase antibodies were comparable to CA125 values, and the combination of these molecules revealed potentially high diagnostic utility [108].

Anti-laminin-1 antibodies were associated with recurrent miscarriage, and increased levels were described in infertile women, as well as in patients with endometriosis. However, this biomarker has not yet passed a diagnostic threshold in current medical practice [109,110].

Two studies conducted by Mathur et al. [111] and Odukoya et al. [112] identified a potential correlation between IgG and endometriosis. IgG antibodies were identified in 56% of affected women and 5% of healthy controls. Another investigation highlighted the presence of IgG in 33% of cases, and of IgM in 27% of them [113].

Given the results of these studies, some autoantibodies could be considered as noninvasive biomarkers for the diagnosis of endometriosis, but further research is necessary.

#### 6.1.7. Proteomics, Metabolomics, and Genomics: The Novel Perspectives of Noninvasive Biomarkers

Proteomics is a new and challenging perspective in the field of noninvasive biomarkers for early detection of endometriosis, which includes all the protein “fingerprints” used for endometriosis diagnosis. Despite promising results, these technologies need better standardization and are cost and time-intensive [12].

Long et al. [113] collected serum samples from several affected women and compared them to controls, in order to detect different protein fingerprints of this disease, by using MALDI-TOF–MS. Their results indicated that 13 protein peaks were over-expressed, and five protein peaks were down-regulated in the affected group compared to healthy subjects.

A further study analyzed distinct patterns of serum proteins in 90 endometriotic women, using SELDI-TOF MS. Following surgical intervention, 51 out of 90 patients were diagnosed with endometriosis, while 39 were unaffected. The researchers concluded that a unique combination of proteins, with molecular weights ranging between 2000 and 20,000 Da made a difference between affected women and controls. The sensitivity of this technique was 81.3%, with a specificity of 60.3% [114]. According to Zheng et al. [115], a proteomic fingerprint model including three peptide peaks showed a sensitivity and specificity of 91.4% and 95%, respectively, for the detection of endometriosis, when compared to controls. In an independent cohort, this combination of peptides revealed a sensitivity of 89.3% and a specificity of 90%.

Wang and collab. [116] illustrated a panel of five protein peaks with a specificity of 90% and a sensitivity of 91.7% for endometriosis, and Jing et al. [117] highlighted two protein peaks with specificity and sensitivity of 97% and 87%, respectively.

Given the inflammatory nature of the disease, inflammation-related proteins have also been investigated for their utility as noninvasive biomarkers. Plasma levels of AXIN1 and ST1A1 were analyzed using ELISA in both affected subjects and healthy controls. AXIN1 and ST1A1 had higher values in endometriosis when compared to healthy controls, regardless of the anatomical location of the lesions [118]. AXIN1 is a promising protein that should be further investigated as a biomarker for endometriosis diagnosis.

Signorile and coworkers [119] used 2D gel analysis in order to describe the potential of two proteins (serum albumin and complement C3 precursor) as diagnostic markers for endometriosis. Their technique was easily reproducible, and their results indicated a sensitivity/specificity for albumin and the complement C3 of 83.3%/83.3% and 58.1%/100%, respectively. In conclusion, this study confirmed the statistical significance of the differential expression for these two proteins in endometriotic women with respect to unaffected individuals. The same authors conducted, in the year 2014, a study comprising 120 women with endometriosis and 20 healthy controls, in order to highlight their serum levels of Zn-alpha2-glycoprotein. After performing ELISA, they observed that the serum levels of this protein were significantly increased in the endometriosis group than in healthy women [120]. In the issue of this observation, the analysis of Zn-alpha2-glycoprotein levels in the serum could become an innovative noninvasive diagnostic test for endometriosis.

Additionally, several studies regarding the metabolome of endometriotic patients have been conducted. Dutta et al. [121] included 22 endometriotic patients and 23 healthy women. They revealed elevated values of Lactate, 3-Hydroxybutyrate, Alanine, Glycerophosphatidylcholine, Valine, Leucine, Threonine, 2-Hydroxybutyrate, Lysine, and Succinic acid in the serum samples of affected women. Furthermore, lower values of glucose, L-Isoleucine, and L-Arginine were discovered in the endometriosis group.

Another study, conducted by Vouk et al. [122] analyzed plasma samples from 40 women with ovarian endometriosis and 52 controls, who underwent laparoscopy. The researchers used electrospray ionization tandem mass spectrometry for more than 140 targeted analytes, including sphingolipids, glycerophospholipids, and acylcarnitines. The results concluded that a model containing hydroxysphingomyelin C16:1 and the ratio between phosphatidylcholine PCaa C36:2 to ether-phospholipid PCae C34:2, had a sensitivity and specificity of 90% and 84.3%, respectively.

In order to establish if the metabolomic profile of affected patients could serve as a noninvasive biomarker for the diagnosis of this pathology, further thorough research is needed.

Genomics may prove an untapped biomarker source with a promising perspective in early diagnosis of endometriosis. Gene-based technologies include cDNA microarray techniques and cDNA hybridization. Recent studies suggested higher plasma concentrations of circulating cell-free cDNA in women with endometriosis [123], in comparison to healthy individuals.

The mitochondrial genome represents another unexplored and potentially promising biomarker pool [108]. Creed et al. [124], highlighted two Mitochondrial DNA (mtDNA) deletions (1.2 and 3.7 kb) which could serve as noninvasive biomarkers.

#### 6.1.8. miRNAs 

MicroRNAs (miRNA) are non-coding RNAs with approximately 21–25 nucleotides and represent major modulators of gene expression in a wide range of pathologies, including endometriosis [125]. miRNA profiling is usually achieved by microarray, followed by qRT-PCR validation. Due to their high stability in the biological fluids and tissue specificity, miRNAs might prove desirable molecules for the diagnosis of endometriosis and other pathologies [126], giving a whole new dimension to the noninvasive diagnosis of endometriosis [127].

Despite several studies on miRNA in endometriosis, the results are still equivocal, with no single miRNA or panel of biomarkers showing enough specificity and sensitivity for this pathology, but with promising results.

MiR-200 is a miRNAs family consisting of three members: miR-200a, miR-200b, and miR-141, which have been thoroughly studied for their dysregulations associated with endometriosis. A study performed by Ohlsson et al. [127] revealed a sensitivity of 84.4% and a specificity of 66.7% in endometriotic patients.

Although not highly specific, miR-20 can be potentially used as a biomarker in early diagnosis of endometriosis. Furthermore, it has been proven that miR-20a targets TGF-β and Il-8, and its down-regulation leads to increased concentrations of these pro-inflammatory molecules [128].

MiR-199a revealed a specificity and sensitivity of 78.33% and 76%, respectively, for endometriosis, according to the results of Wang and collab. [129]. Furthermore, this molecule targets CLIC4 and VCL, inducing a high infiltration proclivity of endometrial cells [129]. Kozomara et al. [130] quantified miR-145 in the peripheral blood of endometriotic patients and compared the values with those from healthy controls. They observed that miR-145 is up-regulated in advanced stages of endometriosis, but in early stages, its down-regulated values are not suitable for diagnosis.

Wang and collab [129] analyzed various miRNAs in 765 serum samples from both cases and healthy controls. Their results showed that miR-199a and miR-122 were up-regulated in affected women in comparison to controls, while miR-9, mi-R-141, mi-R-145, miR-542-3p registered decreased values in endometriosis.

miRNA-200 family is one of the most studied groups of miRNAs molecules, and Rekker et al. [131] concluded that miR-200a-3p and miR-141-3p are more sensitive and specific for endometriosis and that these members of miR-200 family are down-regulated in endometriosis, in comparison with the control group.

As previously mentioned, the results regarding the role of miRNAs as a diagnostic tool in endometriosis are controversial and still equivocal. While several studies support the considerable potential of miRNAs as biomarkers for early stages diagnosis, others revealed a modest specificity of these molecules. Nisenblat et al. [132] determined 49 miRNAs, differentially expressed in endometriotic women. A panel formed by miR-155, miR574-3p, and miR139-3p revealed a sensitivity of 83% and a specificity of only 51%.

Vanhie et al. [133] considered 42 miRNAs, out of which only one panel, namely: hsa-miR-125b-5p, hsa-miR-28-5p and hsa-miR-29a-3p revealed a moderate sensitivity and modest specificity of 78% and 37%, respectively.

In Table 1, we summarized the previous studies illustrating the sensitivity and specificity of different dysregulated miRNAs in endometriosis.

Long non-coding RNAs (lncRNAs) represent a class of molecules with a length of more than 200 nucleotides. They lack the ability of protein-coding [137]. Wang W. et al. [138] applied genome-wide profiling in order to investigate serum lncRNAs differentially expressed in endometriosis women in comparison to healthy controls. They concluded that these molecules presented significant deregulated expressions. Using the PCR quantification method, the authors revealed that 16 lncRNAs were clearly able to distinguish endometriosis from healthy patients—the levels of NR_038452 and ENST00000393610 lncRNA molecules were significantly higher in the serum of affected women, while ENST00000529000, ENST00000482343, ENST00000544649, NR_038395, NR_033688, and ENST00000465368 were decreased in endometriosis. Furthermore, they highlighted an optimal panel of lncRNAs (NR_038395, NR_038452, ENST00000482343, ENST00000544649, and ENST00000393610) able to differentiate between affected and healthy patients. This study concluded that the lncRNAs panel with the highest potential for early detection of endometriosis contains five molecules, namely: NR_038395, NR_038452, ENST00000482343, ENST00000544649, and ENST00000393610.

TC0101441 is a lncRNA molecule with high potential as a biomarker for endometriosis diagnosis, severity, and recurrence prediction. A recent study [139] analyzed the expression of TC0101441 in the serum of affected women versus healthy controls. The authors observed increased TC0101441 levels in the exosomes derived from the serum of endometriotic patients, and a positive and statistically significant correlation between these levels and infertility, chronic pelvic pain, and recurrence of the disease.

### 6.2. Urinary Biomarkers in Endometriosis 

Given its ease of access and fluid composition, urine is one of the most widely used biological samples. As such, the increased accuracy of a urinary test could ideally establish the diagnosis of endometriosis without the need for undergoing invasive surgical interventions. Similar to serum, urinary biomarkers have been measured by a wide range of protein detection methods and have been reported both alone and in combinations [7].

Several urinary peptides and proteins were analyzed for their potential as biomarkers, but further studies are required, with larger sample sizes, in a more diverse population [140]. Creatinine-corrected soluble fms-like tyrosine kinase (sFlt-1) is one of these promising urinary biomarkers for endometriosis. Cho et al. [79] described increased urinary sFlt-1 levels in endometriotic patients. Furthermore, serum sFlt-1 and urinary sFlt-1 levels, corrected for creatinine, were increased in women with endometriosis stage I and II.

Cytokeratin-19 (CK19) is another molecule that could be used in the detection of endometriosis. Kuessel et al. [141] measured urinary levels of CK19 by ELISA on a cohort of 76 women. The study concluded that there was no significant correlation between urinary levels of CK19 and endometriosis. Tokushige et al. [142] used an immunoblot technique to prove that CK19 is expressed only in the urine of women with confirmed endometriosis while absent in the urine of healthy women.

Matrix metalloproteinases (MMP) have been proven to be involved in the pathophysiology of endometriosis, and thus, several authors studied their level in affected women. A panel composed of MMP-2, MMP-9, and MMP-9/neutrophil gelatinase-associated lipocalin was found to increase in 33 subjects with confirmed endometriosis when compared to controls [143].

Using MALDI-TOF MS (matrix-assisted laser desorption/ionization mass spectrometry (MALDI MS) and time-of-flight analyzer (TOF)), differential peptide profiles have been described in the urine of endometriotic women, compared to healthy subjects. Tokushige and colleagues [144], revealed a 12-fold higher expression of five proteins in affected women by combining MALDI-TOF with two-dimensional polyacrylamide gel electrophoresis and El-Kasti et al. [145] highlighted a 3280.9 Da periovulatory peptide that differentiates women with all stages of endometriosis from healthy subjects (sensitivity 82% and specificity 88%).

Cho et al. [146] used western blot and ELISA to investigate urinary proteins of patients with endometriosis. They concluded that 22 protein spots were differentially expressed in the urine of endometriotic women, and one of them was urinary vitamin D-binding protein (VDBP). The conclusion was that urinary VDBP corrected for creatinine (VDBP-Cr) was significantly elevated in patients with endometriosis. The sensitivity of this biomarker was 58%, and the specificity, 76%. When combined with serum CA-125 levels, the authors observed that the positive predictive value was not significantly increased compared to CA-125 alone.

Potlog-Nahari et al. [147] included 40 affected women, with histologically confirmed endometriosis, and 22 healthy controls. They measured the urinary level of VEGF corrected for creatinine. The results of this study revealed no significant difference between urinary levels of VEGF in women with and without endometriosis. Furthermore, these values have not significantly varied between groups according to the phase of the menstrual cycle, hence the unlikeliness that urinary VEGF could serve as a biomarker for endometriosis. No other authors investigated this molecule with respect to endometriosis.

Chen and coworkers [148] were the first to identify histone 4 as a potential urinary biomarker in endometriosis. The authors observed an elevated level of histone 4 in the urine of endometriotic women with a sensitivity and specificity of 70% and 80%, respectively. Considering this research as a key stone, further studies could be conducted in order to validate and strengthen the hypothesis of Chen et al.

In Table 2, we summarize the studies illustrating the main noninvasive biomarkers assessed for endometriosis.

## 7. Conclusions

Despite its benign character, endometriosis remains a debilitating disease that interferes with the quality of life of millions of women. The main symptoms: pelvic pain, infertility, and severe dysmenorrhea are nonspecific, and thus, invariably lead to a long-term delay in establishing the appropriate diagnosis. An invasive procedure, surgery, followed by a histopathological exam, represents the gold standard for diagnosis. As such, literature review proved to be a saga of noninvasive biomarkers, with researchers focusing their efforts into developing noninvasive putative biomarkers for this pathology in hope for promising perspectives on early diagnosis.

Various lines of evidence support the potential role of many biomolecules or panels of biomolecules, but, as of yet, none of them have the test proof sensitivity and specificity. Therefore, they can only complement the diagnosis of this pathology, in conjunction with imaging techniques or laparoscopic surgery.

Inflammatory cytokines proved to be the most non-suitable candidates for noninvasive diagnosis of endometriosis, despite the ability of several panels of cytokines to discriminate between affected and non-affected patients. Similarly, no single miRNA or lncRNA was considered as a sole noninvasive biomarker. According to various authors, a panel of miRNAs or lncRNAs could be more conducive indicators of this gynecological pathology.

The latest technologies consisting of proteomics, metabolomics, and genomics that investigate a complete panel of molecules or a profile of genes could evolve into the gold standard diagnostic tool and thus eliminate invasive laparoscopies.

Furthermore, increased knowledge of the pathophysiologic mechanisms of endometriosis is crucial for a more expedient and accurate diagnosis, improving the overall health-related quality of life.

## Figures and Tables

**Table 1:** Studies reporting the sensitivity and specificity of dysregulated miRNAs in endometriosis.

Author, Reference	Dysregulated miRNA	Specificity	Sensitivity
**Ohlsson et al., 2009** [127]	miR-200a, miR-200b, and miR-141	66,7%	84,4%
**Jia et al., 2013** [134]	miR-22 miR-17-5p miR-20a	90% 80% 90%	90% 60% 60%
**Wang et al., 2013** [129]	miR-145 miR-122 miR-199a miR-141-5p	96% 76% 76% 96%	70% 80% 78,3% 71,7%
**Suryavanshi et al., 2013** [135]	miR-195, miR-16, miR-191	60%	88%
**Rekker et al., 2015** [131]	miR-200a-3p miR-200b-3p miR-141-3p	70.8% 90.6% 70.8%	71.9% 70.8% 71.9%
**Cosar et al., 2016** [136]	miR-125b	96%	100%
**Nisenblat et al., 2019** [132]	miR-155, miR574-3p and miR139-3p	51%	83%
**Vanhie et al., 2019** [133]	hsa-miR-125b-5p, hsa-miR-28-5p and hsa-miR-29a-3p	37%	78%

**Table 2:** Studies assessing the main noninvasive biomarkers for endometriosis.

Author, Reference	Source	Investigated Biomarkers
**Agic et al, 2008** [62]	Serum	CA-125, CCR-1, miRNA, MCP-1
**Borrelli et al., 2014** [71]	Serum	IL-8, MCP-1, RANTES
**Chen et al., 2012** [104]	Serum	Serum pigment epithelium-derived factor
**Chen et al., 2019** [148]	Urine	Histone 4
**Chmaj-Wierzchowska et al., 2015** [99]	Serum	Urocortin, ghrelin, leptin
**Cho et al., 2007** [79]	Serum, Urine	TNF-α, urinary sFlt-1
**Cho et al., 2012** [146]	Urine	Vitamin D-binding protein
**Choi et al., 2019** [82]	Serum	IL-32, IL-6, IL-10, IL-1β, TNF-α, CA-125
**Cosar et al., 2016** [136]	Serum	miR-125b
**Creed et al., 2019** [124]	Serum	mtDNA
**Darai et al., 2003** [77]	Serum	IL-6, IL-8, TNF-α
**Dutta et al., 2012** [91]	Serum	Lactate, 3-Hydroxybutyrate, Alanine, Glycerophosphatidylcholine, Valine, Leucine, Threonine, 2-Hydroxybutyrate, Lysine, Succinic acid
**Gajbhiye et al., 2012** [106]	Serum	Autoantibodies against tropomodulin 3 (TMOD3), tropomyosin 3 (TPM3), stomatin-like protein 2 (SLP2)
**Gmyrek et al., 2005** [149]	Serum	MCP-1
**Gungor et al., 2009** [150]	Serum	Leptin
**Huang et al., 2004** [151]	Serum	MMP2
**Jia et al., 2013** [134]	Serum	miR-22 miR-17-5p miR-20a
**Jing et al., 2008** [117]	Plasma	Proteomics-mass spectrometry
**Kocbek et al., 2015** [66]	Serum	Glycodelin-A
**Kuessel et al., 2014** [141]	Urine	CK19
**Liu et al., 2007** [139]	Plasma	Proteomics – mass spectrometry
**Malin et al., 2019** [118]	Serum	AXIN1
**Martinez et al., 2007** [70]	Serum	IL-6
**May et al., 2010** [12]	Serum	FGF-2, angiogenin
**Mihalyi et al., 2010** [61]	Serum	CA-125, IL-8, TNF-α
**Morin et al., 2005** [152]	Peripheral blood	Macrophage migration inhibitory factor
**Mosbah et al., 2016** [65]	Serum	ICAM-1, glycodelin
**Nabeta et al., 2009** [108]	Serum	Anti-α-enolase antibodies
**Nasiri et al., 2017** [87]	Serum	Malondialdehyde (MDA)
**Nisenblat et al., 2019** [132]	Serum	miR-155, miR574-3p, and miR139-3p
**Ohata et al., 2008** [76]	Seum	IL-8
**Ohlsson et al., 2009** [127]	Serum	miR-200a, miR-200b, and miR-141
**Othman et al., 2008** [70]	Serum	Il-6, MCP-1, IFN-γ IL-2, IL-8, IL-15
**Ozhan et al., 2014** [105]	Serum	Syntaxin-5, anti-endometrial antibody
**Philippoussis et al., 2004** [95]	Serum	IGFBP3, EGF
**Pizzo et al., 2002** [75]	Serum	IL-8
**Potlog-Nahar.i, 2004** [147]	Urine	VEGF
**Qui et al., 2019** [139]	Serum	lncRNA
**Reis et al., 2012** [68]	Serum	Activin A, follistatin
**Rekker et al., 2015** [125]	Serum	miR-200a-3p miR-200b-3p miR-141-3p
**Seeber et al., 2008** [80]	Serum	TNF-α
**Sengul et al., 2014** [97]	Serum	Nesfatin-1
**Socolov et al., 2010** [73]	Serum	IL-6, IL-8, IL-1, CA-125, TNF
**Steff et al., 2004** [94]	Serum	IGF-1, IGFBP3
**Suryavanshi et al., 2013** [135]	Serum	miR-195, miR-16, miR-191
**Tokmak et al., 2011** [98]	Serum	Urocortin
**Tokushige et al., 2011** [144]	Urine	CK19
**Becker et al., 2010** [143]	Urine	MMP-2, MMP-9
**Tuten et al., 2014** [64]	Serum	Copeptin, CA-125, C-reactive protein, CA-15-3, CA-19-9
**Vanhie et al., 2019** [133]	Serum	hsa-miR-125b-5p, hsa-miR-28-5p and hsa-miR-29a-3p
**Vigano et al., 2003** [69]	Serum	ICAM-1
**Vodolazkaia et al., 2012** [63]	Serum	VEGF, CA-125, Annexin V, glycodelin
**Wang et al., 2013** [129]	Serum	miR-145 miR-122 miR-199a miR-141-5p
**Wang et al., 2016** [138]	Serum	lncRNA
**Xavier et al., 2006** [78]	Serum	TNF-α, VEGF

## Abbreviations

**BMI**	Body mass index
**C3**	Complement
**CK 19**	Cytokeratin 19
**COX-2**	Cyclooxygenase 2
**CRP**	C-reactive protein
**DNA**	Deoxiribonucleic acid
**EGF**	Endothelial growth factor
**EGFR**	Endothelial growth factor receptor
**ELISA**	Enzyme-linked immunosorbent assay
**FGF**	Fibroblast growth factor
**GM-CSF**	Granulocyte macrophage colony-stimulating factor
**HOXA**	Homebox A
**ICAM**	Intercellular adhesion molecule
**IFN**	Interferon
**IGF**	Insulin-like growth factor
**Il**	Interleukin
**iNKT**	Invariant natural killer T cells
**lncRNA**	Long non-coding ribonucleic acid
**MCP-1**	Monocyte chemoattractant protein 1
**MIF**	Macrophage inhibitory factor
**MMP**	Matrix metalloproteinase
**miRNA**	Micro ribonucleic acid
**mtDNA**	Mitochondrial DNA
**NK**	Natural killer
**NF-KB**	Nuclear factor-kappa beta
**PDGF**	Platelet-derived growth factor
**PG**	Prostaglandins
**PGE2**	Prostaglandin E2
**PK-1**	Prokinetitsin 1
**RANTES**	Regulated on Activation, Normal T Cell Expressed and Secreted
**ROS**	Reactive oxygen species
**SOD**	Superoxide dismutase
**sFlt1**	Soluble fms-like tyrosine kinase
**TNF**	Tumor-necrosis factor
**TSP-1**	Thrombospondin 1
**VDBP**	Urinary vitamin D binding protein
**VEGF**	Vascular endothelial growth factor
**VEGFR**	Vascular endothelial growth factor receptor
**Wnt/b-catenin**	Wingless-type mouse mammary tumor virus integration site family

## References

[1] Bratila E., Comandasu D.-E., Coreleuca C., Cirstoiu M., Bohiltea R., Mehedintu C., Vladareanu S., Berceanu C. (2016). Gatrointestinal Symptoms in Endometriosis Correlated With the Disease Stage. https://www.researchgate.net/publication/312552546_Gastrointestinal_symptoms_in_endometriosis_correlated_with_the_disease_stage.

[2] Giudice L.C., Kao L.C. (2004). Endometriosis. Lancet.

[3] Moga M.A., Balan A., Dimienescu O.G., Burtea V., Dragomir R.M., Anastasiu V.C. (2019). Circulating miRNAs as biomarkers for endometriosis and endometriosis-related ovarian cancer—An overview. J. Clin. Med..

[4] Bulletti C., Coccia M.E., Battistoni S., Borini A. (2010). Endometriosis and infertility. J. Assist. Reprod. Genet..

[5] Argawal S.K., Chapron C., Giudice L.C., Laufer M.R., Lyland N., Missmer S.A., Singh S.S., Taylor H.S. (2019). Clinical diagnosis of endometriosis: A call to action. Am. J. Obstet. Gynecol..

[6] Nnoaham K.E., Hummelshoj L., Webster P., D’Hooghe T., de Cicco Nardone F., de Cicco Nardone C., de Cicco Nardone C., Jerkinson C., Kennedy S.H., Zondervan K.T. (2011). Impact of endometriosis on quality of life and work productivity: A multicenter study across ten countries. Fertil. Steril..

[7] Fassbender A., Burney R.O., D’Hooghe T., Giudice L. (2015). Update on biomarkers for the detection of endometriosis. BioMed Res. Int..

[8] Hadfield R., Mardon H., Barlow D., Kennedy S. (1996). Delay in the diagnosis of endometriosis: A survey of women from the USA and the UK. Hum. Reprod..

[9] Ahn S.H., Singh V., Tayade C. (2017). Biomarkers in endometriosis: Challenges and opportunities. Fertil. Steril..

[10] Irungu S., Mavrelos D., Worthington J., Blyuss O., Saridogan E., Timms J.F. (2019). Discovery of non-invasive biomarkers for the diagnosis of endometriosis. Clin. Proteom..

[11] Biomarkers Definitions Working Group (2001). Biomarkers and surrogate endpoints: Preferred definitions and conceptual framework. Clin. Pharmacol. Ther..

[12] May K.E., Conduit-Hulbert S.A., Villar J., Kirtley S., Kennedy S.H., Becker C.M. (2010). Peripheral biomarkers of endometriosis: A systematic review. Hum. Reprod. Update.

[13] Halme J., Hammond M.G., Hulka J.F., Raj S.G., Talbert L.M. (1984). Retrograde menstruation in healthy women and in patients with endometriosis. Obstet. Gynecol..

[14] Lagana A.S., Vitale S.G., Salmeri F.M., Triolo O., Ban Frangez H., Vrtacnik-Bokal E., Stojanovska L., Apostolopoulos V., Granese R., Sofo V. (2017). Unus pro omnibus, omnes pro uno: A novel, evidence-based, unifying theory for the pathogenesis of endometriosis. Med. Hypotheses.

[15] Pluchino N., Taylor H.S. (2016). Endometriosis and stem cell trafficking. Reprod. Sci..

[16] Lagana A.S., Salmeri F.M., Vitale S.G., Triolo O., Gotte M. (2017). Stem cell trafficking during endometriosis: May epigenetics play a pivotal role?. Reprod. Sci..

[17] Eggers J.C., Martino V., Reinbold R., Schafer S.D., Kiesel L., Starzinski-Powitz A., Schuring A.N., Kemper B., Greve B., Gotte M. (2016). microRNA miR-200b affects proliferation, invasiveness and stemness of endometriotic cells by targeting ZEB1, ZEB2 and KLF4. Reprod. Biomed. Online.

[18] Marki G., Bokor A., Rigo J., Rigo A. (2017). Physical pain and emotion regulation as the main predictive factors of health-related quality of life in women living with endometriosis. Hum. Reprod..

[19] Lagana A.S., La Rosa V.L., Rapisarda A.M.C. (2017). Anxiety and depression in patients with endometriosis: Impact and management challenges. Int. J. Women Health..

[20] Zheng W., Cao L., Zheng X., Yuanyuan M., Liang X. (2018). Anti-Angiogenic Alternative and complementary medicines for the treatment of endometriosis: A review of potential molecular mechanisms. Evid. Based Complement. Alternat. Med..

[21] Lousse J.C., Van Langendonckt A., Defrere S., Gonzalez Ramos R., Colette S. (2012). Peritoneal endometriosis is an inflammatory disease. Frontiers Biosci..

[22] Gazvani R., Templeton A. (2002). Peritoneal environment, cytokines and angiogenesis in the pathophysiology of endometriosis. Reprod..

[23] Van Langendonckt A., Casanas-Roux F., Donnez J. (2002). Oxidative stress and peritoneal endometriosis. Fertil. Steril..

[24] Oral E., Olive D.L., Arici A. (1996). The peritoneal environment in endometriosis. Hum. Reprod. Update.

[25] Wu M.H., Shoji Y., Chuang P., Tsai S. (2007). Endometriosis: Disease pathophysiology and the role of prostaglandins. Expert Rev Mol Med..

[26] Sugino N., Karube-Harada A., Taketani T., Sakata A., Nakamura Y. (2004). Withdrawal of ovarian steroids stimulates prostaglandin F2-alpha production through nuclear factor-kappaB activation via oxygen radicals in human endometrial stromal cells: Potential relevance to menstruation. J. Reprod. Dev..

[27] Banu S.K., Lee J., Speights V.O., Starzinski-Powitz A., Arosh J.A. (2008). Cyclooxygenase-2 regulates survival, migration, and invasion of human endometriotic cells through multiple mechanisms. Endocrinol.

[28] Wu M.H., Wang C.A., Lin C.C., Chen L.-C., Chang W.-C., Tsai S.-J. (2005). Distinct regulation of cyclooxygenase-2 by interleukin-1 beta in normal and endometriotic stromal cells. J. Clin. Endocrinol. Metab..

[29] Lagana A.S., Garzon S., Gotte M., Vigano P., Franchi M., Ghezzi F., Martin D.C. (2019). The pathogenesis of endometriosis: Molecular and cell biology insights. Int. J. Mol. Sci..

[30] Laird S.M., Tuckerman E.M., Cork B.A., Li T.C. (2000). Expression of nuclear factor kappa B in human endometrium; role in the control of interleukin 6 and leukaemia inhibitory factor production. Mol. Hum. Reprod..

[31] Page M., Tuckerman E.M., Li T.C., Laird S.M. (2002). Expression of nuclear factor kappa-B components in human endometrium. J. Reprod. Immunol..

[32] King A.E., Critchley H., Kelly R.W. (2001). The NF-kappaB pathway in human endometrium and first trimester decidua. Mol. Hum. Reprod..

[33] González Ramos R., Van Langendonckt A., Defrère S., Lousse J.C., Colette S., Devoto L., Donnez J. (2010). Involvement of the nuclear factor-kappaB (NF-kappa B) pathway in the pathogenesis of endometriosis. Fertil Steril.

[34] Lousse J.C., Van Langendonckt A., González Ramos R., Defrère S., Renkin E., Donnez J. (2008). Increased activation of nuclear factor-kappa B (NF-kappa B) in isolated peritoneal macrophages of patients with endometriosis. Fertil. Steril..

[35] Agarwal A., Gupta S., Sikka S. (2006). The role of oxidative stress in endometriosis. Curr. Opin. Obstet. Gynecol..

[36] Zeller J.M., Henig I., Radwanska E., Dmowski W.P. (1987). Enhancement of human monocyte and peritoneal macrophage chemiluminescence activities in women with endometriosis. Am. J. Reprod. Immunol. Microbiol..

[37] Portz D.M., Elkins T.E., White R., Warren J., Adadevoh S., Randolph J. (1991). Oxygen free radicals and pelvic adhesion formation: Blocking oxygen free radical toxicity to prevent adhesion formation in an endometriosis model. Int. J. Fertil..

[38] Wang Y., Sharma R.K., Falcone T., Goldberg J., Agarwal A. (1997). Importance of reactive oxygen species in the peritoneal fluid of women with endometriosis or idiopathic infertility. Fertil. Steril..

[39] Polak G., Koziol-Montewka M., Gogacz M., Blaszkowska I., Kotarski J. (2001). Total antioxidant status of the peritoneal fluid in infertile women. Eur. J. Obstet. Gynecol. Reprod. Biol..

[40] Wu M.H., Hsiao K.Y., Tsai S.J. (2015). Endometriosis and possible inflammation markers. Gynecol. Minim. Invasive Ther..

[41] Lagana A.S., Salmeri F.M., Ban Frangez H., Ghezzi F., Vrtacnik-Bokal E., Granese R. (2019). Evaluation of M1 and M2 macrophages in ovarian endometriomas from women affected by endometriosis at different stages of the disease. Gynecol. Endocrinol..

[42] Capobianco A., Monno A., Cottone L., Venneri M.A., Biziato D., Di Puppo F., Ferrari S., De Palma M., Manfredi A.A., Rovere-Querini P. (2011). Proangiogenic Tie2(+) macrophages infiltrate human and murine endometriotic lesions and dictate their growth in a mouse model of the disease. Am. J. Pathol..

[43] Lagana A.S., Ghezzi F., Vetvicka V. (2019). Endometriosis and risk of ovarian cancer: What do we know?. Arch. Gynecol. Obstet..

[44] Wu M.Y., Yang J.H., Chao K.H., Hwang J.L., Yang Y.S., Ho H.N. (2000). Increase in the expression of killer cell inhibitory receptors on peritoneal natural killer cells in women with endometriosis. Fertil. Steril..

[45] Lagana A.S., Triolo O., Salmeri F.M., Granese R., Palmara V.I., Ban Frangez H., Vrtcnik Bokal E., Sofo V. (2016). Natural Killer T cell subsets in eutopic and ectopic endometrium: A fresh look to a busy corner. Arch. Gynecol. Obstet..

[46] Ho H.N., Wu M.Y., Chao K.H., Der Chen C., Chen S.U., Yang Y.S. (1997). Peritoneal interleukin-10 increases with decrease in activated CD4+ T lymphocytes in women with endometriosis. Hum. Reprod..

[47] Nie M.-F., Xie Q., Wu Y.-H., He H., Zou L.-J., She X.-L., Wu X.-Q. (2018). Serum and Ectopic Endometrium from Women with Endometriosis Modulate Macrophage M1/M2 Polarization via the Smad2/Smad3 Pathway. J. Immun. Res..

[48] Kralickova M., Vetvicka V. (2015). Immunological aspects of endometriosis: Review. Ann. Transl. Med..

[49] Osuga Y., Hirota Y., Hirata T., Takamura M., Urata Y., Harada M., Izumi G., Fujii T., Koga K. (2016). Th2 Cells and Th17 cells in the development of endometriosis—Possible roles of interleukin-4 and interleukin-17A. J. Endometr. Pelvic Pain Dis..

[50] Asante A., Taylor R.N. (2011). Endometriosis: The role of neuroangiogenesis. Ann. Rev. Physiol..

[51] Hanahan D., Folkman J. (1996). Patterns and emerging mechanisms of the angiogenic switch during tumorigenesis. Cell.

[52] Ahn S.H., Edwards A.K., Singh S.S., Young S.L., Lessey B.A., Tayade C. (2015). Il-17A contributes to the pathogenesis of endometriosis bu triggering proinflammatory cytokines and angiogenic growth factors. J. Immunol..

[53] Gupta M.K., Qin R.Y. (2003). Mechanism and its regulation of tumor-induced angiogenesis. World J. Gastroenterol..

[54] Strieter R.M., Polverini P.J., Kunkel S.L., Arenberg D.A., Burdick M.D., Kasper J., Dzuiba J., Van Damme J., Walz A., Marriott D. (1995). The functional role of the ELR motif in CXC chemokine-mediated angiogenesis. J. Biol. Chem..

[55] Volpert O.V., Fong T., Koch A.E., Peterson J.D., Waltenbaugh C., Tepper R.I., Bouck N.P. (1998). Inhibition of angiogenesis by interleukin 4. J. Exp. Med..

[56] Torisu H., Ono M., Kiryu H., Furue M., Ohmoto Y., Nakayama J., Nishioka Y., Sone S., Kuwano M. (2000). Macrophage infiltration correlates with tumor stage and angiogenesis in human malignant melanoma: Possible involvement of TNFalpha and IL-1alpha. Int. J. Cancer.

[57] Donnez J., Smoes P., Gillerot S., Casanas-Roux F., Nisolle M. (1998). Vascular endothelial growth factor (VEGF) in endometriosis. Hum. Reprod..

[58] Machado D.E., Rodrigues-Baptista K.C., Perini J.A. (2016). Soares de Moura, R. Euterpe oleracea extract (Açaí) is a promising novel pharmacological therapeutic treatment for experimental endometriosis. PLoS ONE.

[59] Thambisetty M., Lovestone S. (2010). Blood-based biomarkers of Alzheimers disease: Challenging but feasible. Biomark. Med..

[60] Mol B.W.J., Bayram N., Lijmer J.G., Wiegerinck M.A.H.M., Bongers M.Y., Van der Veen F., Bossuyt P. (1998). The performance of CA-125 measurement in the detection of endometriosis: A meta-analysis. Fertil. Steril..

[61] Mihalyi A., Gevaert O., Kyama C.M., Simsa P., Pochet N., De Smet F., De Moor B., Meuleman C., Billen J., Blanckaert N. (2010). Non-invasive diagnosis of endometriosis based on a combined analysis of six plasma biomarkers. Hum. Reprod..

[62] Agic A., Djalali S., Wolfler M.M., Halis G., Diedrich K., Hornung D. (2008). Combination of CCR1 mRNA, MCP1, and CA125 measurements in peripheral blood as a diagnostic test for endometriosis. Reprod. Sci..

[63] Vodolazkaia A., El-Aalamat Y., Popovic D. (2012). Evaluation of a panel of 28 biomarkers for the non-invasive diagnosis of endometriosis. Hum. Reprod..

[64] Ozhan E., Kokcu A., Yanik K., Gunaydin M. (2014). Investigation of diagnostic potentials of nine different biomarkers in endometriosis. Eur. J. Obstet. Gynecol..

[65] Mosbah A., Nabiel Y., Khashaba E. (2016). Interleukin-6, intracellular adhesion molecule-1, and glycodelin A levels in serum and peritoneal fluid as biomarkers for endometriosis. Obstet. Gynecol..

[66] Kocbek V., Vouk K., Bersinger N.A., Mueller M.D., Lanišnik Rižner T. (2015). Panels of Cytokines and Other Secretory Proteins as Potential Biomarkers of Ovarian Endometriosis. J. Mol. Diagn..

[67] Pretice Crapper E. (2016). Clinical Biomarkers for the Noninvasive Diagnosis of Endometriosis. http://hdl.handle.net/11375/20495.

[68] Reis F.M., Luisi S., Abrão M.S., Rocha A.L.L., Viganò P., Rezende C.P., Florio P., Petraglia F. (2012). Diagnostic value of serum activin A and follistatin levels in women with peritoneal, ovarian and deep infiltrating endometriosis. Hum. Reprod..

[69] Vigano P., Infantino M., Lattuada D., Lauletta R., Ponti E., Somigliana E., Vignali M., DiBlasio A.M. (2003). Intercellular adhesion molecule-1 (ICAM-1) gene polymorphisms in endometriosis. Mol. Hum. Reprod..

[70] Othman E.E.R., Hornung D., Salem H.T., Kalifa E.A., El-Metwally T.H., Al-Hendy A. (2008). Serum cytokines as biomarkers for nonsurgical prediction of endometriosis. Eur. J. Obstet. Gynecol. Repd. Biol..

[71] Borrelli G.M., Abrão M.S., Mechsner S. (2014). Can chemokines be used as biomarkers for endometriosis? A systematic review. Hum. Reprod..

[72] Martinez S., Garrido N., Coperias J.L., Pardo F., Desco J., Garcia-Velasco J.A., Simon C., Pellicer A. (2007). Serum interleukin-6 levels are elevated in women with minimal-mild endometriosis. Hum. Reprod..

[73] Socolov R., Butureanu S., Angioni S., Sindilar A., Boiculese V.L., Cozma L., Socolov D. (2010). The value of serological markers in the diagnosis and prognosis of endometriosis: A prospective case-control study. Eur. J. Obstet. Gynecol. Reprod. Biol..

[74] Kalu E., Sumar N., Giannopoulos T., Patel P., Croucher C., Sherriff E., Bansal A. (2007). Cytokine profiles in serum and peritoneal fluid from infertile women with and without endometriosis. J. Obstet. Gynaecol. Res..

[75] Pizzo A., Salmeri F.M., Ardita F.V., Sofo V., Tripepi M., Marsico S. (2002). Behaviour of cytokine levels in serum and peritoneal fluid of women with endometriosis. Gynecol. Obstet. Investig..

[76] Ohata Y., Harada T., Miyakoda H., Taniguchi F., Iwabe T., Terakawa N. (2008). Serum interleukin-8 levels are elevated in patients with ovarian endometrioma. Fertil. Steril..

[77] Darai E., Detchev R., Hugol D., Quang N.T. (2003). Serum and cyst fluid levels of interleukin (IL) -6, IL-8 and tumour necrosis factor-alpha in women with endometriomas and benign and malignant cystic ovarian tumours. Hum. Reprod..

[78] Xavier P., Belo L., Beires J., Rebelo I., Martinez-de-Oliveira J., Lunet N., Barros H. (2006). Serum levels of VEGF and TNF-a and their association with C-reactive protein in patients with endometriosis. Arch. Gynecol. Obstet..

[79] Cho S.H., Oh Y.J., Nam A., Kim H.Y., Park J.H., Kim J.H., Cho D.J., Lee B.S. (2007). Evaluation of Serum and Urinary Angiogenic Factors in Patients with Endometriosis. Am. J. Reprod. Immunol..

[80] Seeber B., Sammel M.D., Fan X., Gerton G.L., Shaunik A., Chittams J., Barnhart K.T. (2008). Panel of markers can accurately predict endometriosis in a subset of patients. Fertil. Steril..

[81] Steff A.M., Gagné D., Pagé M., Rioux A., Hugo P., Gosselin D. (2004). Insulin-like growth factor-1, soluble tumor necrosis factor receptor-1 and angiogenin in endometriosis patients. Am. J. Reprod. Immunol..

[82] Choi Y.S., Kim S., Oh Y.S., Cho S., Hoon Kim S. (2019). Elevated serum interleukin-32 levels in patients with endometriosis: A cross-sectional study. Am. J. Reprod. Immunol..

[83] Kikuchi Y., Ishikawa N., Hirata J., Imaizumi E., Sasa H., Nagata I. (1993). Changes of peripheral blood lymphocyte subsets before and after operation of patients with endometriosis. Acta Obstet. Gynecol. Scand..

[84] Tuten A., Kucur M., Imamoglu M., Kaya B., Acikgoz A.S., Yilmaz N., Ozturk Z., Oncul M. (2014). Copeptin is associated with the severity of endometriosis. Arch. Gynecol. Obstet..

[85] Carvalho L.F., Samadder A.N., Agarwal A., Fernandes L.F., Abrao M.S. (2012). Oxidative stress biomarkers in patients with endometriosis: Systematic review. Arch. Gynecol. Obstet..

[86] Scutiero G., Iannone P., Bernardi G., Bonaccorsi G., Spadaro S., Volta C.A., Greco P., Nappi L. (2017). Oxidative stress and endometriosis: A systematic review of the literature. Oxidative Med. Cell Longev..

[87] Nasiri N., Moini A., Eftekhari-Yazdi P., Karimian L., Salman-Yazdi R., Arabipoor A. (2017). Oxidative stress statues in serum and follicular fluid of women with endometriosis. Cell. J..

[88] Verit F.F., Erel O., Celik N. (2008). Serum paraoxonase-1 activity in women with endometriosis and its relationship with the stage of the disease. Hum. Reprod..

[89] Jackson L.W., Schisterman E.F., Dey-Rao R., Browne R., Armstrong D. (2005). Oxidative stress and endometriosis. Hum. Reprod..

[90] Turkyilmaz E., Yildirim M., Cendek B.D., Baran P., Alisik M., Dalgaci F., Yavuz A.F. (2016). Evaluation of oxidative stress markers and intra-extracellular antioxidant activities in patients with endometriosis. Eur. J. Obstet. Gynecol. Reprod. Biol..

[91] Prieto L., Quesada J.F., Cambero O., Pacheco A., Pellicer A., Codoceo R., Garcia-Velasco J.A. (2012). Analysis of follicular fluid and serum markers of oxidative stress in women with infertility related to endometriosis. Fertil. Steril..

[92] Andrisani A., Dona G., Brunati A.M., Clari G., Armanini D., Ragazzi E., Ambrosini G., Bordin L. (2014). Increased oxidation-related glutathionylation and carbonic anhydrase activity in endometriosis. Reprod. Biomed. Online.

[93] Gurgan T., Bukulmez O., Yarali H., Tanir M., Akyildiz S. (1999). Serum and peritoneal fluid levels of IGF I and II and insulin-like growth binding protein-3 in endometriosis. J. Reprod. Med..

[94] Steff A.M., Gagne D., Page M., Hugo P., Gosselin D. (2004). Concentration of soluble intercellular adhesion molecule-1 in serum samples from patients with endometriosis collected during the luteal phase of the menstrual cycle. Hum. Reprod..

[95] Philippoussis F., Gagne D., Hugo P., Gosselin D. (2004). Concentrations of alpha-fetoprotein, insulin-like growth factor binding protein-3, c-erbB-2, and epidermal growth factor in serum of patients with endometriosis. J. Soc. Gynecol. Investig..

[96] Garcia-Galiano D., Navarro V.M., Gaytan F., Tena-Sempere M. (2010). Expanding roles of NUCB2/nesfatin-1 in neuroendocrine regulation. J. Mol. Endocrinol..

[97] Sengul O., Dilbaz B., Halici Z., Ferah I., Cadirci E., Yilmaz F. (2014). Decreased serum nesfatin-1 levels in endometriosis. Eur. J. Obstet. Gynecol. Reprod. Biol..

[98] Tokmak A., Ugur M., Tonguc E., Var T., Moraloglu O., Ozaksit G. (2011). The value of urocortin and CA-125 in the diagnosis of endometrioma. Arch. Gynecol. Obstet..

[99] Chmaj-Wierzchowska K., Kampioni M., Wilczak M., Sajdak S., Opala T. (2015). Novel markers in the diagnostics of endometriomas: Urocortin, ghrelin and leptin or leukocytes, fibrinogen, and CA-125?. Taiwan. J. Obstet. Gynecol..

[100] Florio P., Reis F.M., Torres P.B., Calonaci F., Toti P., Bocchi C., Linton E.A., Petraglia F. (2007). Plasma urocortin levels in the diagnosis of ovarian endometriosis. Obstet. Gynecol..

[101] Mohamed M.L., El Behery M.M., Mansour S.A.E.-A. (2013). Comparative study between VEGF-A and CA-125 in diagnosis and follow-up of advanced endometriosis after conservative laparoscopic surgery. Arch. Gynecol. Obstet..

[102] Bourlev V., Iljasova N., Adamyan L., Larsson A., Olovsson M. (2010). Signs of reduced angiogenic activity after surgical removal of deeply infiltrating endometriosis. Fertil. Steril..

[103] Szubert M., Suzin J., Duechler M., Szuławska A., Czyz M., Kowalczyk-Amico K. (2014). Evaluation of selected angiogenic and inflammatory markers in endometriosis before and after danazol treatment. Reprod. Fertil. Dev..

[104] Chen L., Fan R., Huang X., Xu H., Zhang X. (2012). Reduced levels of serum pigment epithelium-derived factor in women with endometriosis. Reprod. Sci..

[105] Ozhan E., Kokcu A., Yanik K., Gunaydin M. (2014). Investigation of diagnostic potentials of nine different biomarkers in endometriosis. Eur. J. Obstet. Gynecol. Reprod. Biol..

[106] Gajbhiye R., Sonawani A., Khan S., Suryawanshi A., Kadam S., Warty N., Raut V., Khole V. (2012). Identification and validation of novel serum markers for early diagnosis of endometriosis. Hum. Reprod..

[107] Yi Y.-C., Wang S.-C., Chao C.-C., Su C.-L., Lee Y.-L., Chen L.-Y. (2010). Evaluation of serum autoantibody levels in the diagnosis of ovarian endometrioma. J. Clin. Lab Anal..

[108] Nabeta M., Abe Y., Kagawa L., Haraguchi R., Kito K., Ueda N., Sugita A., Yokoyama M., Kusanagi Y., Ito M. (2009). Identification of anti-α-enolase autoantibody as a novel serum marker for endometriosis. Proteom. Clin. Appl..

[109] Inagaki J., Matsuura E., Kaihara K., Kobayashi K., Yasuda T., Nomizu M., Sugiura-Ogasawara M., Katano K., Aoki K. (2001). IgG antilaminin-1 autoantibody and recurrent miscarriages. Am. J. Reprod. Immunol..

[110] Inagaki J., Sugiura-Ogasawara M., Nomizu M., Nakatsuka M., Ikuta K., Suzuki N., Kaihara K., Kobayashi K., Yasuda T., Shoenfeld Y. (2003). An association of IgG anti-laminin-1 autoantibodies with endometriosis in infertile patients. Hum. Reprod..

[111] Mathur S., Garza D.E., Smith L.F. (1990). Endometrial autoantigens eliciting immunoglobulin (Ig) G, IgA, and IgM responses in endometriosis. Fertil. Steril..

[112] Odukoya O.A., Wheatcroft N., Weetman A.P., Cooke I.D. (1995). The prevalence of endometrial immunoglobulin G antibodies in patients with endometriosis. Hum. Reprod..

[113] Long X., Jinag P., Zhou L., Zhang W. (2013). Evaluation of novel serum biomarkers and the proteomic differences of endometriosis and adenomyosis using MALDI-TOF–MS. Arch. Gynecol Obstet..

[114] Wölfler M.M., Schwamborn C., Otten D., Hornung D., Liu H., Rath W. (2009). Mass spectrometry and serum pattern profiling for analyzing the individual risk for endometriosis: Promising insights?. Fertil. Steril..

[115] Zheng N., Pan C., Liu W. (2011). New serum biomarkers for detection of endometriosis using matrix-assisted laser desorption/ionization time-of-flight mass spectrometry. J. Int. Med. Res..

[116] Wang L., Zheng W., Mu L., Zhang S.-Z. (2008). Identifying biomarkers of endometriosis using serum protein fingerprinting and artificial neural networks. Int. J. Gynaecol. Obstet..

[117] Jing J., Qiao Y., Suginami H., Taniguchi F., Shi H., Wang S. (2008). Two novel serum biomarkers for endometriosis screened by surface-enhanced laser desorption/ionization time-of-flight mass spectrometry and their change after laparoscopic removal of endometriosis. Fertil. Steril..

[118] Malin E., Bodil R., Gunnar E., Bodil O. (2019). AXIN1 in Plasma or Serum Is a Potential New Biomarker for Endometriosis. Int. J. Mol. Sci..

[119] Signorile P.G., Baldi A. (2015). Supporting evidences for potential biomarkers of endometriosis detected in peripheral blood. Data Brief..

[120] Signorile P.G., Baldi A. (2014). Serum Biomarker of Endometriosis. J. Cell Physiol..

[121] Dutta M., Joshi M., Srivastava S., Lodh I., Chakravarty B., Chaudhury K. (2012). A metabonomics approach as a means for identification of potential biomarkers for early diagnosis of endometriosis. Mol. Biosyst..

[122] Vouk K., Hevir N., Ribic-Pucelj M., Haarpaintner G., Scherb H., Osredkar J., Moller G., Prehn C., Lanisnik Rizner T., Adamski J. (2012). Discovery of phosphatidylcholines and sphingomyelins as biomarkers for ovarian endometriosis. Hum. Reprod..

[123] Zachariah R., Schmid S., Radpour R., Buerki N., Fan A.X.-C., Hahn S., Holzgreve W., Zhong X.Y. (2009). Circulating cell free DNA as a potential biomarker for minimal and mild endometriosis. Reprod. Biomed. Online.

[124] Creed J., Maggrah A., Reguly B., Harbottle A. (2019). Mitochondrial DNA deletions accurately detect endometriosis in symptomatic females of child-bearing age. Biomark. Med..

[125] Bartel D.P. (2004). MicroRNAs: Genomics, biogenesis, mechanism, and function. Cell.

[126] Weber J.A., Baxter D.H., Zhang S., Huang D.Y., Huang K.H., Lee M.J., Galas D.J., Wang K. (2010). The microRNA spectrum in 12 body fluids. Clin. Chem..

[127] Ohlsson Teague E.M.C., Print C.G., Hull M.L. (2009). The role of microRNAs in endometriosis and associated reproductive conditions. Hum. Reprod. Update.

[128] Zhao M., Tang Q., Wu W., Xia Y., Chen D., Wang X. (2014). miR-20a contributes to endometriosis by regulating NTN4 expression. Mol. Biol. Rep..

[129] Wang W.T., Zhao Y.N., Han B.W., Hong S.J., Chen Y.Q. (2013). CirculatingMicroRNAsIdentifiedinaGenome-Wide Serum MicroRNA Expression Analysis as Noninvasive Biomarkers for Endometriosis. J. Clin. Endocrinol. Metab..

[130] Kozomara A., Birgaoanu M., Griffiths-Jones S. (2019). miRBase: From microRNA sequences to function. Nucleic Acids Res..

[131] Rekker K., Saare M., Roost A.M., Kaart T., Sõritsa D., Karro H., Sõritsa A., Simón C., Salumets A., Peters M. (2015). Circulating miR-200-family micro-RNAs have altered plasma levels in patients with endometriosis and vary with blood collection time. Fertil. Steril..

[132] Nisenblat V., Sharkey D.J., Wang Z., Evans S.F., Healey M., Ohlsson Teague E.M.C., Print C.G., Robertson S.A., Hull M.L. (2019). Plasma miRNAs display limited potential as diagnostic tools for endometriosis. Clin. Endocrinol. Metab..

[133] Vanhie A., Peterse D.O.D., Beckers A., Cuellar A., Fassbender A., Meuleman C., Mestdagh P., D’Hooghe T. (2019). Plasma miRNAs as biomarkers for endometriosis. Hum. Reprod..

[134] Jia S.Z., Yang Y., Lang J., Sun P., Leng J. (2013). Plasma miR-17–5p, miR-20a and miR-22 are down-regulated in women with endometriosis. Hum. Reprod..

[135] Suryawanshi S., Vlad A.M., Lin H.M., Mantia-Smaldone G., Laskey R., Lee M., Lin Y., Donnellan N., Klein-Patel M., Lee T. (2013). Plasma MicroRNAs as novel biomarkers for endometriosis and endometriosis-associated ovarian cancer. Clin. Cancer Res..

[136] Cosar E., Mamillapalli R., Ersoy G.S., Cho S., Seifer B., Taylor H.S. (2016). Serum microRNAs as diagnostic markers of endometriosis: A comprehensive array-based analysis. Fertil. Steril..

[137] Zhang X.-Y., Zheng L.-W., Li C.-J., Xu Y., Zhou X., Fu L.-I., Li D.-D., Sun L.-T., Zhang D., Cui M.-H. (2019). Dysregulated Expression of Long Noncoding RNAs in Endometriosis. Crit. Rev. Eukaryot. Expr..

[138] Wang W.-T., Sun Y.-M., Huang W., He B., Zhao Y.-N., Chen Y.-Q. (2016). Genome-wide long non-coding RNA Analysis identified circulating LncRNAs as novel non-invasive diagnostic biomarkers for gynecological disease. Sci. Rep..

[139] Qiu J., Zhang X., Ding Y., Hua K. (2019). 1856 circulating exosomal long noncoding RNA-TC0101441 as a non-invasive biomarker for the prediction of endometriosis severity and recurrence. J. Minim. Invasive Gynecol..

[140] Gueye N.A., Stanhiser J., Valentine L., Kotlyar A., Goodman L., Falcone T. (2017). Biomarkers for Endometriosis in saliva, urine, and peritoneal fluid. Biomark. endometr..

[141] Kuessel C., Jaeger-Lamsky A., Pateisky P., Rossberg N., Schulz A., Schmitz A.A.P., Staudigl C., Wenzl R. (2014). Cytokeratin-19 as a biomarker in urine and in serum for the diagnosis of endometriosis—A prospective study. Gynecol. Endocrinol..

[142] Hawkins S.M., Creighton C.J., Han D.Y., Zariff A., Anderson M.L., Gunaratne P.H., Matzuk M.M. (2011). Functional microRNA involved in endometriosis. Molec Endocrinol..

[143] Becker C.M., Louis G., Exarhopoulos A., Mechsner S., Ebert A.D., Zurakowski D., Moses M.A. (2010). Matrix metalloproteinases are elevated in the urine of patients with endometriosis. Fertil. Steril..

[144] Tokushige N., Markham R., Crossett B., Ahn S.B., Nelaturi V.L., Khan A., Fraser I.S. (2011). Discovery of a novel biomarker in the urine in women with endometriosis. Fertil. Steril..

[145] El-Kasti M.M., Wright C., Fye H.K.S., Roseman F., Kessler B.M., Becker C.M. (2011). Urinary peptide profiling identifies a panel of putative biomarkers for diagnosing and staging endometriosis. Fertil. Steril..

[146] Cho S., Choi Y.S., Yim S.Y., Yang H.I., Jeon Y.E., Lee K.E., Kim H.Y., Seo S.K., Lee B.S. (2012). Urinary vitamin D-binding protein is elevated in patients with endometriosis. Hum. Reprod..

[147] Potlog-Nahari C., Stratton P., Winkel C., Widra E., Sinaii N., Connors S., Nieman L.K. (2004). Urine vascular endothelial growth factor-A is not a useful marker for endometriosis. Fertil. Steril..

[148] Chen X., Liu H., Sun W., Guo Z., Lang J. (2019). Elevated urine histone 4 levels in women with ovarian endometriosis revealed by discovery and parallel reaction monitoring proteomics. J. Proteom..

[149] Gmyrek G.B., Sozanski R., Jerzak M., Chrobak A., Wickiewicz D., Skupnik A., Sierazka U., Fortuna W., Gabrys M., Chelmonska-Syta A. (2005). Evaluation of monocyte chemotactic protein-1 levels in peripheral blood of infertile women with endometriosis. Eur. J. Obstet. Gynecol. Reprod. Biol..

[150] Gungor T., Kanat-Pektas M., Karayalcin R., Mollamahmutoglu L. (2009). Peritoneal fluid and serum leptin concentrations in women with primary infertility. Arch. Gynecol. Obstet..

[151] Huang H., Hong H., Tan Y., Sheng J. (2004). Matrix metalloproteinase 2 is associated with changes in steroids hormones in the sera and peritoneal fluid of patients with endometriosis. Fertil. Steril..

[152] Morin M., Bellehumeur C., Therriault M.J., Metz C., Maheux R., Akoum A. (2005). Elevated levels of macrophage migration inhibitory factor in the peripheral blood of women with endometriosis. Fertil. Steril..

